# [Retracted] Downregulation of miR‑30a is associated with proliferation and invasion via targeting MEF2D in cervical cancer

**DOI:** 10.3892/ol.2023.14168

**Published:** 2023-11-27

**Authors:** Jing Zhao, Bo Li, Chuqiang Shu, Yun Ma, Yingping Gong

Oncol Lett 14: 7437–7442, 2017; DOI: 10.3892/ol.2017.7114

Following the publication of this paper, it was drawn to the Editor's attention by a concerned reader that certain of the Transwell invasion assay data shown in Fig. 2C on p. 7439 were strikingly similar to data that had appeared in different form in different articles published by different authors at different research institutes.

Owing to the fact that some of the data in the above article had already been published, or were under consideration for publication, prior to its submission to *Oncology Letters*, the Editor has decided that this paper should be retracted from the Journal. The authors were asked for an explanation to account for these concerns, but the Editorial Office did not receive a reply. The Editor apologizes to the readership for any inconvenience caused.

